# Biological Surface Layer Formation on Bioceramic Particles for Protein Adsorption

**DOI:** 10.3390/biomimetics9060347

**Published:** 2024-06-08

**Authors:** Reo Kimura, Daichi Noda, Zizhen Liu, Wanyu Shi, Ryota Akutsu, Motohiro Tagaya

**Affiliations:** Department of Materials Science and Bioengineering, Nagaoka University of Technology, Kamitomioka 1603-1, Nagaoka 940-2188, Japan

**Keywords:** hydroxyapatite, Ca-deficient hydroxyapatite, carbonate-substituted hydroxyapatite, amorphous silica particle, Cl-containing amorphous silica particle, non-apatitic layer, surface modification, protein immobilization, highly ordered structure, biomineralization

## Abstract

In the biomedical fields of bone regenerative therapy, the immobilization of proteins on the bioceramic particles to maintain their highly ordered structures is significantly important. In this review, we comprehensively discussed the importance of the specific surface layer, which can be called “non-apatitic layer”, affecting the immobilization of proteins on particles such as hydroxyapatite and amorphous silica. It was suggested that the water molecules and ions contained in the non-apatitic layer can determine and control the protein immobilization states. In amorphous silica particles, the direct interactions between proteins and silanol groups make it difficult to immobilize the proteins and maintain their highly ordered structures. Thus, the importance of the formation of a surface layer consisting of water molecules and ions (i.e., a non-apatitic layer) on the particle surfaces for immobilizing proteins and maintaining their highly ordered structures was suggested and described. In particular, chlorine-containing amorphous silica particles were also described, which can effectively form the surface layer of protein immobilization carriers. The design of the bio-interactive and bio-compatible surfaces for protein immobilization while maintaining the highly ordered structures will improve cell adhesion and tissue formation, thereby contributing to the construction of social infrastructures to support super-aged society.

## 1. Introduction

The population in the world is aging rapidly, and urgent action should be taken to extend healthy life expectancy. In order to improve this situation, the development of medical technology is necessary, especially drug delivery system (DDS), which has become an efficient bone regenerative therapy technology. When bone growth factor protein drugs are immobilized on DDS carriers, the collapse of the highly ordered protein structure often occurs, resulting in the loss of drug function. To ameliorate the problem, it is important to functionalize the carrier surfaces that could maintain the protein interaction structures. Therefore, in the present review, the structures and characteristics of the representative bioceramic particles such as hydroxyapatite (HA) and amorphous silica were summarized, and the novel protein immobilization techniques on bioceramics such as hydroxyapatite (HA) and amorphous silica hosts were highlighted and investigated by mimicking the steric adsorption states of proteins in vivo. Furthermore, the research on surface functionalization of bioceramic particles for protein immobilization was reviewed to construct the novel research field of surface hydration layer formation and protein interaction states.

## 2. Protein Immobilization Phenomena on Biological Bone

**Figure 1:**
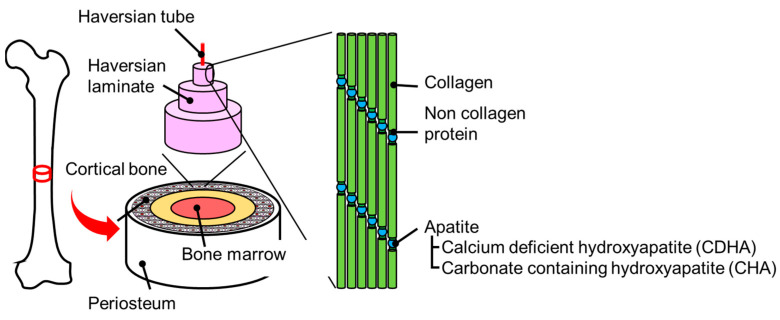
Schematic diagram of the living bone structures. Adapted from Ref. [3].

The illustration of biological bone is shown in Figure 1. In introducing the bone from the outside, the layers of the periosteum, cortical bone, trabecular bone, and bone marrow can be observed. The cortical bone is a thin bone layer called Haber’s lamina that surrounds the channels of blood vessels (i.e., Haber’s canal) like the annual ring structure [1]. Here, the Haber’s lamina is composed of the hybrid bioceramic system of HA with organic/polymeric materials. In particular, collagen is artificially used as the backbone structure and HA fills the structural spaces, and the interfacial collagen-HA binding is effectively attributed to the non-collagen protein medication [2].

The highly ordered structures of proteins are closely related to their functions, and the non-collagen proteins maintain their highly ordered structures and effectively bind with HA, enabling them to perform bone formation and functions. It is known that non-collagen proteins involved in binding to collagen are osteocalcin and osteonectin [2]. The functional molecular part of the non-collagen proteins would be γ-carboxyglutamic acid (Gla) residues with two carboxylate ions, which interact electrostatically with the calcium ions of HA in biological bone mineralization. As a specific behavior, old biological bone is exposed to the acidic environment of osteoclasts, resulting in its dissolution into calcium and phosphate ions. Subsequently, osteoblasts release non-collagen proteins and then precipitate HA on their carboxylate ions as the nucleation sites, indicating the inorganic–organic binding interfaces in vivo. However, there have been few studies that mimic the mechanism of biological bone mineralization, which is important for novel research on the surface modification of bioceramics and the formation of their interactive interfaces with proteins.

## 3. Bioceramic Particles and Surface Function

### 3.1. Hydroxyapatite Particles

#### 3.1.1. Features

**Figure 2:**
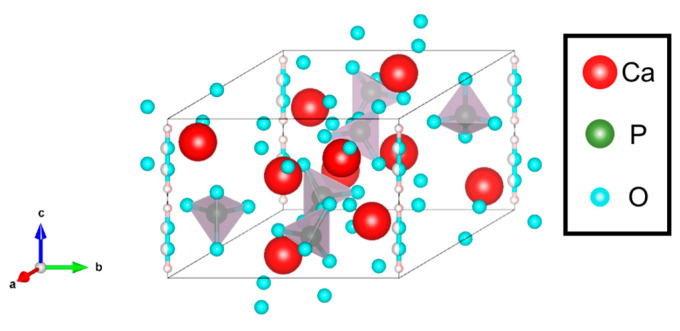
Crystal structure of HA. Here, the grey parts indicate phosphate ions.

HA, which has higher biocompatibility, is one of the bioceramics and the main component of biological bone. For the crystalline structure (Figure 2), HA is hexagonal, the space group is classified into P6_3_/m, and the lattice constants are *a* = 0.94 nm and *c* = 0.68 nm. The ten Ca ions in HA can be divided into two different locations: four Ca ions are Ca(I) (columnar Ca) and are arranged in a single column along the *c*-axis, while the other Ca ions are Ca(II) (axis Ca) and form the equilateral triangles around the *c*-axis at the four corners of the HA unit cell, which are rotated by 60° and stacked along the *c*-axis direction. The hydroxyl groups are stacked and arranged along the *c*-axis direction in the region surrounded by Ca(II), and these Ca(I) and Ca(II) are surrounded by oxygen atom of PO_4_^3−^ [4]. The *a*-plane (*m*-plane) is positively charged with many Ca^2+^ ions, whereas the *c*-plane with many PO_4_^3−^ ions is negatively charged. Moreover, the hydrophilic OH and PO_4_^3−^ in the HA structure form hydrogen bonds with the functional groups of proteins, making it easy to immobilize proteins. Furthermore, HA also has higher ion exchange ability. For example, various elemental ions are substituted and introduced into HA in the biological hard tissues, depending on their concentrations and species, thereby causing changes in the crystal lattice, bone metabolism, and bone formation in vivo. The cationic substitutions with Ca^2+^ and the anionic substitutions with PO_4_^3−^ and OH^−^ of different elemental ions will affect the lattice constant, crystallinity, surface charge, and morphology of HA. Na^+^ and K^+^ are abundant in vivo and also exist in the biological bones, whereas the substitution elements change the lattice parameters of HA due to factors such as the loss of OH^−^ and Ca^2+^ and the increase in ionic diameters. For example, it has been reported that Na^+^ and CO_3_^2−^ (type B) substitutions decrease the *a*-axis and increase the *c*-axis, Cl^−^ substitution increases the *a*-axis and decreases the *c*-axis, and K^+^ substitution decreases the *a*- and *c*-axes [5].

HA has been applied to biomedical devices such as artificial bones and implants and to the chromatographic stationary phase due to its high protein adsorption ability [6,7]. Currently, research is being conducted using HA as a drug delivery carrier due to its biocompatibility and protein adsorption ability [8,9]. HA exists in vivo as the Ca-deficient hydroxyapatite (CDHA) and carbonate-containing hydroxyapatite (CHA) structures [10], which can be artificially synthesized and controlled for biomimicry, and interest in the crystalline phases is growing.

#### 3.1.2. Ca-Deficient Hydroxyapatite

CDHA is a type of apatite, and its chemical formula is shown below.

$${\mathrm{Ca}}_{10-x}{\left({\mathrm{HPO}}_{4}\right)}_{x}{\left({\mathrm{PO}}_{4}\right)}_{6-x}{\left(\mathrm{OH}\right)}_{2-x}$$

**Figure 3:**
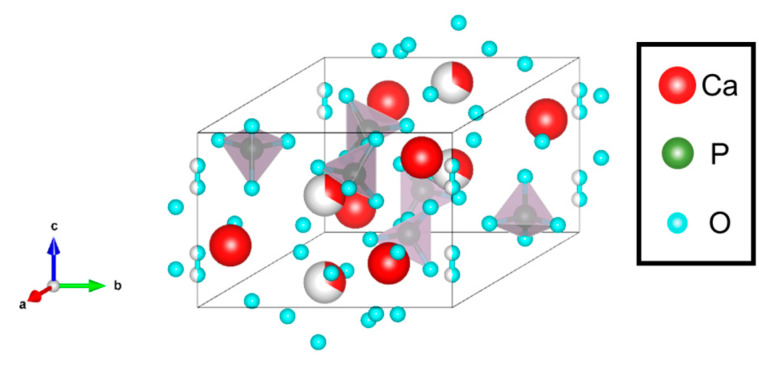
Crystal structure of CDHA. Here, the grey and white parts indicate phosphate ions and Ca(I) sites, respectively.

The Ca/P molar ratio of CDHA is 1.33–1.67, making it more bioabsorbable than the case in the stoichiometric (Ca/P molar ratio of 1.67) HA, which is more similar to the apatite in biological bone. The formation of CDHA is largely related to the dissociation state of the phosphate ions [11,12]. Specifically, it has been reported that HA is formed under a higher proportion of PO_4_^3−^ in solution, whereas CDHA is formed under a higher proportion of HPO_4_^2−^ in solution [13]. With increasing pH during the synthesis, phosphate ions dissociate in the form of H_3_PO_4_→H_2_PO_4_^−^→HPO_4_^2−^→PO_4_^3−^, indicating that HA and CDHA are formed under the higher (pH > 10) and lower (pH 6–8) pH conditions, respectively. The crystal structure of CDHA is shown in Figure 3. The comparison between Figure 2 and Figure 3 indicates that the crystal structure of CDHA is almost identical to that of HA [14]. Specifically, the Ca(I) sites of HA become the vacancies, and part of the PO_4_^3−^ becomes HPO_4_^2−^, so the crystal structure remains almost unchanged compared to the case in HA. It has been reported that the phase transitions occur to form β-phase tricalcium phosphate (β-TCP) at 650–750 °C and to form α-TCP at 1180 °C [15,16]. Moreover, CDHA can also be synthesized by the hydrolysis of α-TCP. These characteristics have significantly promoted the research of CDHA particulation, which is applied to the DDS nanocarriers [17,18]. These reports are related to the CDHA/organic interface hybrid design, suggesting that organic molecules are immobilized on the CDHA surface.

#### 3.1.3. Carbonate-Substituted Hydroxyapatite

**Figure 4:**
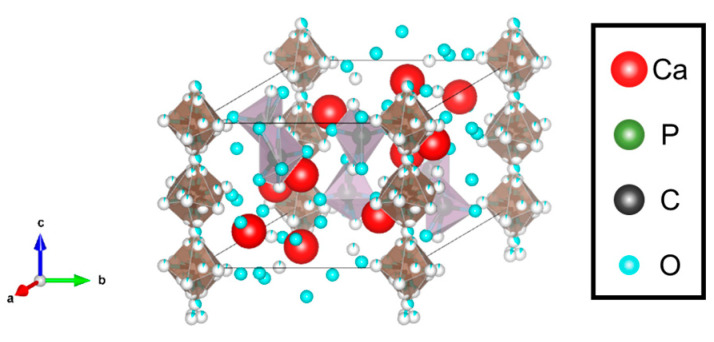
Crystal structure of CHA. Here, the white circles, grey and brown parts indicate oxygen atoms, phosphate ions, and carbonate ions, respectively.

CHA represents all HA structures containing carbonate ions. In this case, the carbonate ions are present through adsorption on the HA surfaces and substitution within the crystal structure. CHA has been known to exhibit higher solubility than HA at lower pH (pH < 7) and has been applied to bone replacement materials [19]. CHA is stable at higher pH (pH > 10). Thus, there are various carbonate ion contents, with biological bone containing approximately 2.3–8 wt% of carbonate ions, and many studies have been conducted in this concentration range [20,21]. The apatite with the carbonate ions substituted within the crystal structures is called carbonate apatite. The carbonate apatite is classified into three types: HA with the carbonate ions substituted with the OH site, as shown in Figure 4, is called type A CHA, and its chemical formula is expressed as follows.

$${\mathrm{Ca}}_{10}{\left({\mathrm{PO}}_{4}\right)}_{6}{\left(\mathrm{OH}\right)}_{2-2x}{\left({\mathrm{CO}}_{3}\right)}_{x}$$

Type A CHA can be synthesized by heating HA at around 1000 °C in a carbon dioxide atmosphere and does not precipitate in most lower-temperature synthesis in the wet process [22]. With increasing CO_3_^2−^ content, an increase in the *a*-axis and a decrease in the *c*-axis of the lattice constants have been observed. When the substitution of CO_3_^2−^ reaches the maximum content of 4.4 wt%, the *a*-axis increases by 0.0025 nm for every 1 wt% increase [23].

The apatite with carbonate ions substituted at the PO_4_^3−^ site is called type B CHA, and its chemical formula is expressed as follows.

$${\mathrm{Ca}}_{10-x/2}{\left({\mathrm{PO}}_{4}\right)}_{6-x}{\left({\mathrm{CO}}_{3}\right)}_{x}{\left(\mathrm{OH}\right)}_{2}$$

Type B CHA can be synthesized by wet process at lower temperatures (≈body temperature at around 40 °C) [24], with the loss of Ca^2+^ and OH^−^ through the substitution of CO_3_^2−^ for the charge compensation, and CO_3_^2−^ is eventually replaced by HPO_4_^2−^. For each 1 wt% substitution of CO_3_^2−^, the *a*-axis decreases by 0.00006 nm and the *c*-axis increases in length, and it has been reported that the substitution amount of CO_3_^2−^ can be as high as 22.2 wt% [25]. The substitutions at both sites are called type AB CHA [26]. CHA and CDHA particles have been investigated as nanocarriers for DDS based on the CHA/organic interface design [27,28].

### 3.2. Amorphous Silica Particles

#### 3.2.1. Features

**Figure 5:**
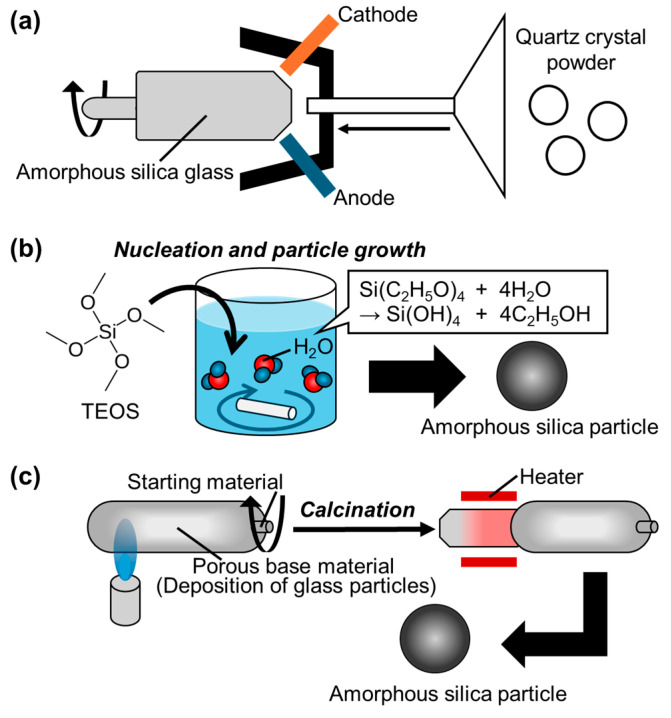
Illustrations of the manufacturing method for the amorphous silica particles, which are (**a**) fusion, (**b**) sol-gel, and (**c**) vapor-axial-phase-deposition methods, respectively. Adapted with permission from Ref. [42]. Copyright 2024 American Chemical Society.

Silica is the generic term for silicon dioxide, in which oxygen and silicon are abundant elements on the earth [29]. Especially, silica particles exist in various forms as crystalline [30] and amorphous states [31]. In particular, amorphous silica particles are usually converted into the high-purity quartz glass, sol, gel and fumed silica, and then have a wide range of applications such as semiconductor encapsulation fillers, liquid crystal display materials [32,33,34,35,36], reinforcing fillers for elastomers and polymers, and key materials in the biomedical field [37,38]. The representative production methods include the melting method [39], the sol-gel method [40] as the liquid-phase method, and the Vapor-Phase-Axial-Deposition (VAD) method as the gas-phase method [41] (Figure 5).

In the melting method to obtain the amorphous silica particles, quartz powder as the starting material is filled in carbon cases and melted using electricity or flames (Figure 5a) [43]. In the electric melting, the particles are melted by electric resistance heating using carbon electrodes [44]. In flame melting, the particles are obtained by melting with an oxygen-hydrogen flame. The sol-gel method is the direct synthesis of the particles using solutions of inorganic and organic metal salts as the starting materials through hydrolysis and condensation polymerization reactions [45,46]. The reactions proceed to form the sol, which then undergoes further reactions to form the gel. The gel is dried at 40–120 °C, then heated and sintered at 900–1200 °C to produce amorphous silica particles (Figure 5b) [47]. The VAD method uses silicon tetrachloride (SiCl_4_) as the starting material and supplies oxygen-hydrogen gas from the burner to obtain the porous matrix, on which the amorphous silica particles formed in the flame are deposited (Figure 5c) [48,49]. Since the process is carried out through a porous matrix, the quality of the synthesized particles can be controlled depending on the sintering process and the presence or absence of the matrix modification process. For example, it is possible to obtain the halogen-containing particles by adding halogen elements to the sintering atmosphere [50,51]. Moreover, the porous matrix is sintered in the atmosphere with a lower moisture content, resulting in the synthesis of particles with lower concentrations of silanol groups and impurities. Thus, the structure and surface properties of the particles depend on the synthetic method, and the selection is important for the applications.

##### Structure and Surfaces

**Figure 6:**
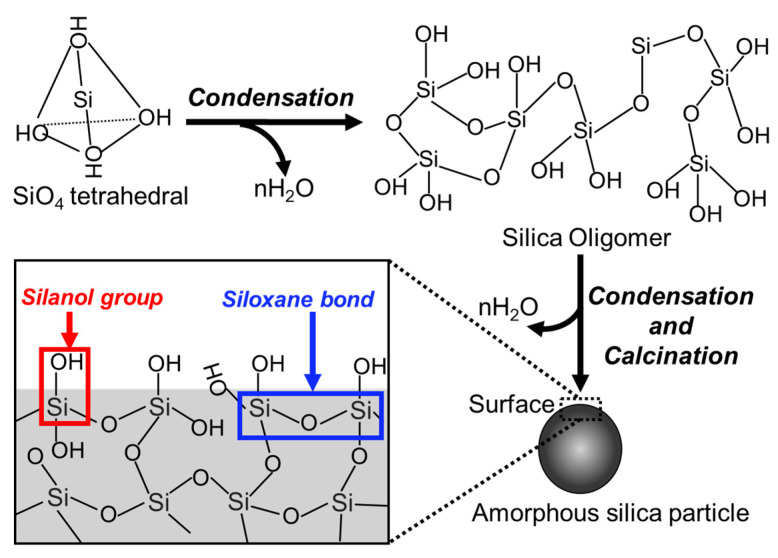
Illustration of the formation mechanism and the surface structure of the amorphous silica particles.

The silica network of the amorphous silica particles consists of the structures in which the SiO_4_ tetrahedral units are irregularly connected to each other through the O atom [52,53,54,55]. There are two main functional groups of the siloxane bond (≡Si–O–Si≡) and the silanol group (≡Si–OH) (Figure 6) [56,57,58].

Siloxane bonds are present from the interior to the top of the particles and are the main chains of the network. Their bond angle range is 120–180° [59,60,61], which allows for a variety of ring structures, including three-, four-, six-, and eight-membered rings [62,63,64,65]. Depending on the bond angles and bond distortions, there are stable or unstable ring structures. Particularly, the planar three-membered ring structure is unstable and is easily hydrolyzed by water molecules [66].

Silanol groups are generally numerous on the amorphous silica particles and can be classified as single, geminal, and triple types when the number of OH groups attached to the Si atom is one, two, or three, respectively [67,68,69]. There are also vicinal silanol groups, which are present with two or more silanol groups in close contact to form hydrogen bonds with each other [70]. Particularly, the presence of silanol groups is an important parameter that determines the physicochemical properties of the particles (e.g., the molecular adsorption sites). Silanol groups interact with polar molecules such as water molecules, which form hydrogen bonds with silanol groups and with other molecules that exhibit hydrogen bond-forming ability [71,72,73,74]. The pH environment causes the different dissociation behaviors of the protons from the silanol groups on the particles, resulting in the changes in surface charge [75,76]. The isoelectric point of amorphous silica particles is around pH 2.0, and in biological fluids at pH 7.4, they have dispersibility due to their electrostatic repulsion of negative charges around −50 mV [77,78]. The higher surface reactivity of silanol groups is an important parameter for applications in biomedical fields [79,80,81,82,83].

#### 3.2.2. Biofunctionalization

The surface functions of amorphous silica particles as bioceramics are given in this section. First, bioglasses (e.g., Na_2_O-CaO-SiO_2_-P_2_O_5_) containing amorphous silica particles (<50 mol%) have been known to show osteoconductive properties [84,85,86,87]. In the biological fluid, Na^+^, Ca^2+^, and phosphate ions are first eluted from the bioglass. Subsequently, the amorphous silica particles on the bioglass form a gel layer, which is the starting point for the adsorption of Ca^2+^ ions. As a result, HA is efficiently precipitated and adhered to biological bone, which has been used as the bone replacement material. Then, amorphous silica particles could induce the production of nutrients for all organs in vivo and have been developed for use in supplemental food products [88,89,90,91]. For example, they promote collagen production in vivo and improve the bonding between collagen and elastin in skin and bone [92,93,94]. Amorphous silica particles have attracted attention as cosmetic ingredients because of their activation of fibroblast functions. For the applications, the protein adsorption form in vivo on surface functions such as silanol groups of amorphous silica particles should be important for the biofunctions in biomedical fields. However, the immobilization states of amorphous silica particles have not yet been clarified. If the immobilization states can be clarified, a wide range of biological and medical applications can be achieved.

#### 3.2.3. Application for Antibody Protein Immobilization

**Figure 7:**
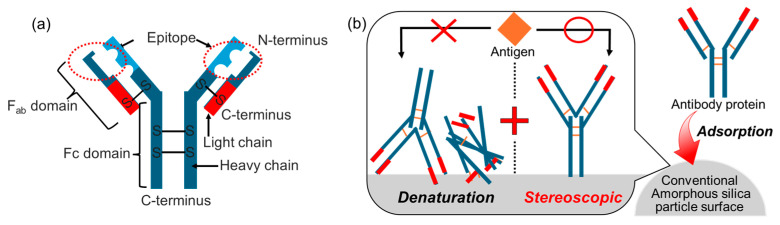
Illustration of the (**a**) antibody protein structure and the (**b**) immobilization state on the conventional amorphous silica particle surfaces.

With the biomedical applications of amorphous silica particles, the antibody test kits and the carriers for the immobilization in immunochromatography can be proposed [95,96]. In the applications, the immobilization of antibody proteins on the particles should be considered to improve their functions. Immunoglobulins are known as antibody proteins with immune functions (Figure 7a) [97,98]. The antibody proteins consist of two heavy chains (H chain: 50–77 kDa) and two light chains (L chain: 25 kDa), which are connected to each other by disulfide bonds and form the Y-shape [99,100,101]. They recognize and specifically bind to antigen (i.e., antigen–antibody reactions) [102,103,104]. The V-shaped part of the Y-shaped arm is termed as F_ab_ (Fragment antigen binding) domain, and the body part is known as the F_c_ (Fragment crystallizable) domain [100]. Immunity is acquired when phagocytes recognize the antibodies that have interacted with antigens through binding of the F_c_ region to phagocyte receptors, resulting in the production of large amounts of antibody proteins in biological fluids [105,106,107]. The variable site of the antibody that recognizes the antigen has been called the antigenic determinant (epitope) and consists of several amino acids [108,109]. The most numerous antibody protein in human blood and biological fluids is immunoglobulin G (IgG), which accounts for about 80% of the total and is approximately [110] 14.5 nm × 8.5 nm × 4.0 nm in size [111]. Immunoassays using these antibody proteins are used for antibody testing [112,113]. Immunochromatography and ELISA are the two main types of immunoassays [114,115,116]. Immunoassays are characterized by the immobilization of antibody proteins on particulate substrates. If the antibody proteins are denatured due to immobilization, it becomes difficult to form binding between the antibody protein and the target antigen, thus adversely affecting the sensitivity of the test (Figure 7b) [117,118]. The orientation of IgG at immobilization is important for immunoassay efficiency. Specifically, it has been reported that by coating glass with n-butyl methacrylate, the adsorbed orientation was controlled by the temperature [119]. Furthermore, in the case of bioceramics, the zirconia particles modified with phosphate groups enable the selective adsorption of amino groups from the F_ab_ and F_c_ regions via electrostatic interactions, suggesting astate where only one F_ab_ region is exposed on the surface [120]. Accordingly, the controlled orientation of the IgG on the amorphous silica particles will be the future challenge. In recent years, the use of amorphous silica particles as immobilization carriers for antibody proteins has been investigated. In conventional particles, the direct interactions with silanol groups on the particles are thought to result in denaturation, leading to lower sensitivity of the immunoassays [121,122]. In fact, there have been cases where IgG has been adsorbed on hydrophilic silica glass, and the subsequent immune response has been investigated. However, the immobilized IgG might have been denatured, as the phagocytic response was completely inhibited [123]. In other words, the challenge for applications such as antibody test kits is to immobilize antibody proteins (IgG) sterically, with a focus on inhibiting direct interactions with the surface of amorphous silica particles.

## 4. The Surface Layer Formed on the Bioceramic Particles

### 4.1. Formation of the Surface Layer in Biological Fluids

**Figure 8:**
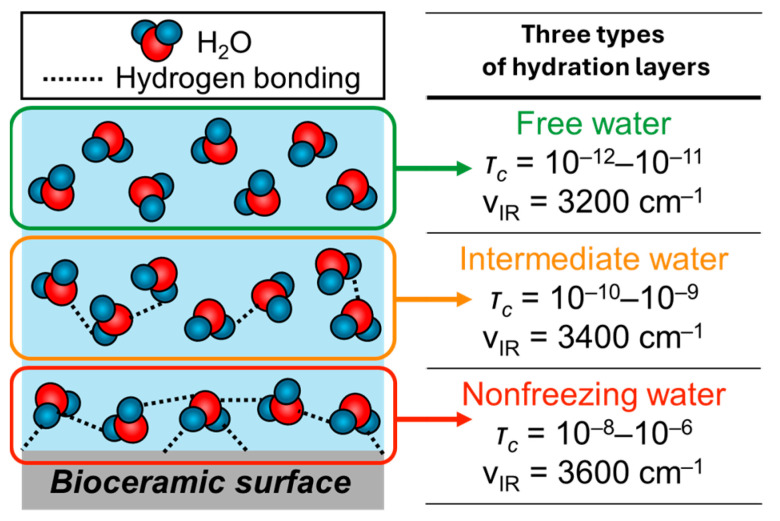
Classification of hydration layers, which were analyzed by relaxation time (τ_c_) of the water molecule motion measured by solid-state NMR and IR adsorption wavenumber ν_IR_. Adapted from Ref. [131].

When considering the immobilization states of antibody proteins on bioceramic particles (e.g., amorphous silica particles), the hydration layer formed at the interfaces between the particles and proteins is very important [124,125]. The hydration layer is formed by the adsorption of water molecules and ions on the bioceramics in biological fluids (Figure 8). Various factors contribute to the formation of the hydration layer, which is mediated between ions and bioceramic surfaces through electrostatic interactions and hydrogen bonding. The hydration layer is composed of three types (i.e., non-freezing water, intermediate water, and free water) [126], which have been mainly investigated in the biopolymers, and this theory has recently also been applied to the bioceramics. Non-freezing water and intermediate water showed direct interactions with bioceramics, reducing the hydrogen bonds between water molecules, resulting in enhanced and maintained dynamics of water molecules with decreasing temperature. In free water, hydrogen bonds are formed between water molecules below 0 °C, and the molecular motion stops and freezing occurs. The differences are due to the strength of interactions acting on the bioceramic surfaces, resulting in different kinetic properties, which can be evaluated by differential scanning calorimetry (DSC) and ^1^H-NMR [127]. According to the DSC measurement, non-freezing water does not freeze at −100 °C, and intermediate water has been reported to be cold recrystallized at lower temperatures below 0 °C with increasing temperature [128]. Free water is the water that melts from its frozen state at 0 °C and interacts weakly with bioceramic surfaces and non-freezing water. The hydration layer shows the recombination behavior from femtoseconds to picoseconds to form the amorphous structures in a very short time and shows the randomness of the liquid on a longer time scale. Therefore, the relaxation times (τ_c_) of non-freezing water, intermediate water, and free water obtained by ^1^H-NMR are 10^−8^–10^−6^, 10^−10^–10^−9^, and 10^−12^–10^−11^ s, respectively. When stronger interactions with the bioceramic surface are observed, the kinetics will be lowered [129]. According to the FT-IR spectra, the absorption bands (*v*_IR_) of the stretching vibrations of hydroxyl groups corresponding to non-freezing water, intermediate water, and free water are observed at 3600 cm^−1^, 3400 cm^−1^ and 3200 cm^−1^, respectively. The *v*_IR_ has been evaluated by the spectral deconvolution and separation techniques [129,130].

### 4.2. Formation of the Surface Layer in Biological Fluids

**Figure 9:**
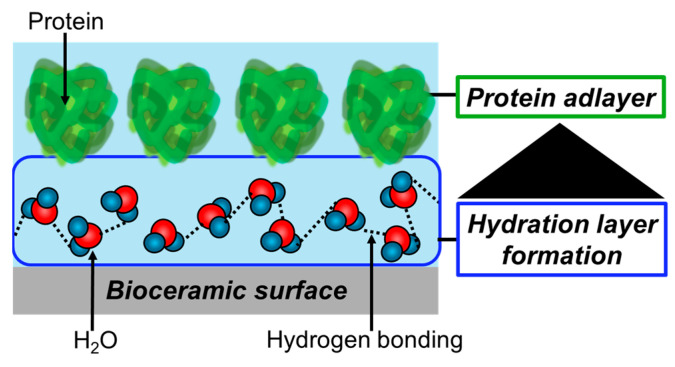
Illustration of the surface layer on bioceramics for protein immobilization.

After the hydration layer is formed on the bioceramics, the protein immobilization layer is formed by recognizing the hydration layer states [132]. In order to consider the protein immobilization states, it is necessary to consider the balance of the hydration layer components composed of non-freezing, intermediate, and free waters (Figure 9). The importance of the balance was supported by the fact that the denaturation of proteins in vivo was observed on the biopolymer without intermediate water in the hydration layer. The relationship between the adsorbed proteins and the hydration layers has recently been applied to bioceramics. For example, our research group has reported that the fibrinogen was sterically adsorbed on the elliptical HA nanocrystals due to the reduced proportion of non-freezing water components [133]. When the silicate ion-containing HA (SiHA) was immersed in biological fluid, the proportion of free water components effectively increased due to the enhanced elution of carbonate ions from the surfaces [134]. Then, the fibrillation of the adsorbed collagen on SiHA was inhibited due to the repulsion between the intermediate waters, thus inducing the steric adsorption of collagen molecules on the surfaces [135]. Therefore, the control of the proportion of hydration layer components is important for the steric adsorption of proteins such as immunoglobulins. Therefore, the sterility of protein immobilization is largely affected by the fraction of each hydration layer component. Furthermore, the immobilization, which maintains the highly ordered structure of the proteins, also affects subsequent cell adhesion. For example, cell adhesion is promoted on the fibronectin adsorbed while maintaining its highly ordered structure on the HA surfaces [136]. Also, the cell-adhesive proteins adsorbed on citric acid-modified HA films, maintaining their higher-order structure, enhanced the growth of the osteoblasts [135]. Thus, the adsorbed state of proteins effectively determines the functions of adhered cells.

### 4.3. Formation of the Non-Apatitic Layer and Protein Immobilization Ability

**Figure 10:**
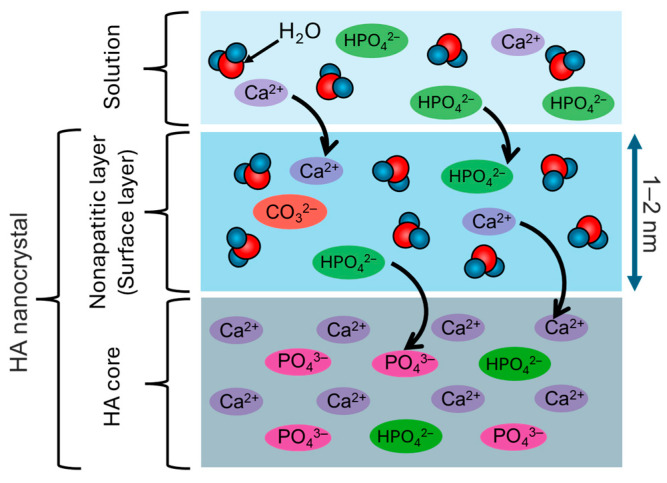
Illustration of the surface layers of HA nanocrystal core in vivo. Adapted from Ref. [131].

The non-apatitic layer has been investigated by FT-IR and NMR spectra, etc. In the FT-IR spectra, the absorption band corresponding to the non-apatitic layer is usually observed at 680−480 cm^−1^, which has been assigned to phosphate ions (e.g., PO_4_^3−^ and HPO_4_^2−^) [137,138]. In terms of the coordination environment and interactions of phosphate ions, 2D solid-state NMR has demonstrated that the HA nanocrystal surfaces were composed of a non-apatitic layer composed of amorphous calcium phosphate (Figure 10) [139,140,141]. The exposed ions on the non-apatitic layer were strongly bound with the O atoms of the H_2_O molecules, partially forming the lewis acidity, indicating the highly reactive surfaces of the non-apatitic layer [142,143]. The ions on the non-apatitic layer were stabilized by the hydration layer, effectively constructing the hydrogen bonding network [143,144]. The non-apatitic layers are involved in the growth and ion exchange of the inner HA nanocrystal core as well as the adsorption of organic ions [145]. For example, the interfacial phenomena between non-apatitic layers and biological fluids are induced in vivo, resulting in the calcification of teeth and bones [146,147,148,149,150]. It is thought that the interfacial phenomena are effectively caused by the ions contained in the non-apatitic layer (i.e., the state of the hydration layer), leading to the protein adsorption states. The ions contained in the non-apatitic layer are important in this sequence of interfacial mechanisms. The silicate and carbonate ions contained in the non-apatitic layer cause the structural defects in the HA nanocrystals, thereby increasing the solubility of the ions in vivo [151,152,153]. The increased solubility of the ions expands the hydrogen bonding network and changes the proportion of the hydration layer component, thus controlling the adsorption states of the protein. Moreover, it has been reported that the presence of ions in the non-apatitic layer on HA nanocrystals facilitates the elution of carbonate ions from the non-apatitic layer, increasing the proportion of free water components in the hydration layer [133]. Therefore, ions in the non-apatitic layer are thought to alter the hydration layer state and affect the protein immobilization state.

The immobilization of proteins on HA nanocrystals is affected by the interactions between the ions in the non-apatitic layer and the functional groups of the proteins. For example, it has been reported that the immobilization of pepsin on the spherical HA nanocrystals modified with cetylpyridinium chloride caused the denaturation due to the Cl^−^ ion diffusion into pepsin [154]. Thus, the ions in the non-apatitic layer can affect the protein immobilization states. Therefore, the non-apatitic layer is thought to be involved in the steric properties of organic molecules and proteins adsorbed on HA nanocrystals, and the protein immobilization states affect the biological functions. For example, the ionic species on the surfaces of HA nanocrystals can control the orientation (i.e., steric state) of adsorption and immobilization of anionic porphyrins [155]. It has also been reported that the adsorbed state of human serum albumin (HSA) on HA nanocrystals with carbonate ions in the non-apatitic layer has a lower proportion of the random coils in the protein secondary structures as compared to the case in HSA in the native state [156]. Furthermore, the steric immobilization of bone morphogenetic protein (BMP-2) and antibody proteins involved in the immunization has also been reported on pure HA nanocrystals [157,158].

As described above, the non-apatitic layer on the HA nanocrystal core surface can sterically immobilize nucleic acids and proteins, which can be applied to the surface modification of protein chromatography columns [159,160]. Therefore, water molecules and ions in the non-apatitic layer would induce the steric immobilization states, although further detailed investigation is needed.

### 4.4. Amorphous Silica Particles for the New Layer Formation

As mentioned above, the formation of a surface layer consisting of water molecules and ions similar to the non-apatitic layer leads to the steric immobilization of proteins. Among the ions contained in the non-apatitic layer, phosphate, Na^+^, and Cl^−^ ions play important roles in biological reactions. Phosphate ions are the main component of the HA nanocrystal, and it is believed that the adsorption states of ions on HA during the nucleation stage are important [5]. Na^+^ ions in the non-apatitic layer provide excellent osteoconductivity to activate osteoblast growth [161]. In fact, there have been reports on the alkaline treatment of the titania surfaces to improve their reactivity with simulated body fluid (SBF). Surface treatment with 10 mM NaOH resulted in the formation of a sodium titanate layer on the surface. The Na^+^ ion contained in this layer caused the exchange with H_3_O^+^ ions in SBF, and the interfaces might be involved in inducing the steric immobilization of proteins [162,163,164,165]. Cl^−^ ions are also contained in the non-apatitic layer, showing excellent osteoconductivity [166]. The inclusion of various ions will improve the reactivity of amorphous silica particles, promote the adsorption of water molecules and ions in biological fluids, and facilitate the steric immobilization of proteins.

In order to form a surface layer composed of water molecules and ions similar to the non-apatitic layer, surface reactivity can be effectively improved by including chlorine in amorphous silica particles. The inclusion of chlorine into silica glass decreases the total amount of silanol groups, subsequently reducing transmission losses and improving the refractive index in the near-infrared light region (λ > 1064 nm) [167]. In the VAD method, chlorine can be contained in amorphous silica particles. In particular, the porous matrix is heat-treated in a mixed atmosphere of chlorine compound vapors with inert gases such as H_2_, Ne, and N_2_ to resultantly achieve inclusion [168]. Here, the two main types of chlorine compound vapors are silicon tetrachloride and chlorine gases. The concentration of chlorine content in silicon tetrachloride gas is higher than that in chlorine gas. In the case of silicon tetrachloride gas, the inclusion concentration of chlorine in the base material can be proportional to 1/4 of the added silicon tetrachloride concentration. It has been reported that the reaction proceeds according to the following chemical equilibrium (Equation (1)).

SiCl_4_ + 3SiO_2_ ⇆ 4SiO_1.5_Cl (1)

where SiO_2_ indicates the porous matrix. Furthermore, when chlorine is contained in amorphous silica particles, the chemical formula is SiO_1.5_Cl [169]. When chlorine gas is used, the inclusion concentration of chlorine in amorphous silica particles is low. Considering this and (Equation (2)), the reaction between the porous matrix and chlorine is required to use silicon tetrachloride. Therefore, the introduction of chlorine components into amorphous silica particles with the chlorine gas are the following two-step reactions.

2Cl_2_ + SiO_2_ ⇆ SiCl_4_ + O_2_ SiCl_4_ + 3SiO_2_ ⇆ 4SiO_1.5_Cl (2)

Here, when chlorine gas is used, the presence of oxygen in the heat treatment makes it difficult to generate silicon tetrachloride according to chemical equilibrium. Thus, it is necessary to reduce the oxygen concentration in the reaction furnace.

**Figure 11:**
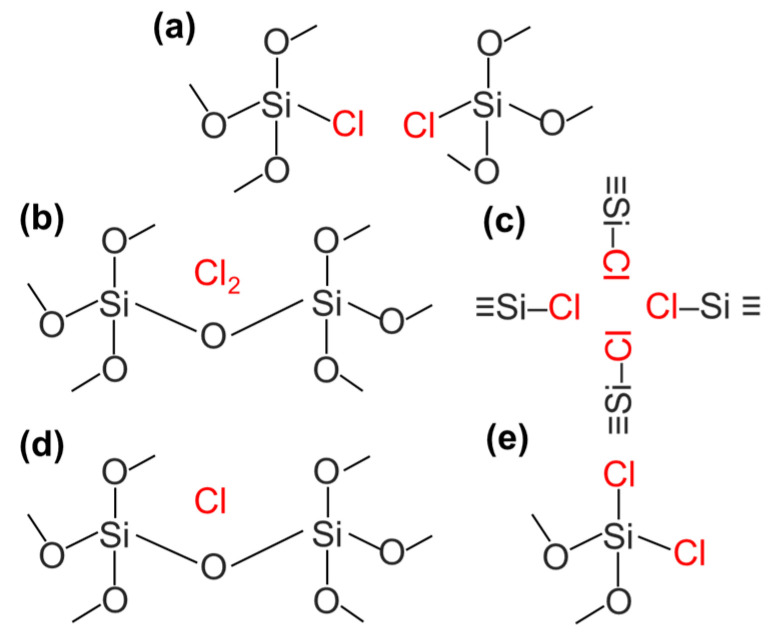
Illustration of the presence states of chlorine (indicated in red) in the amorphous silica particles, which are classified into (**a**) Si–Cl arrangement (main product), (**b**) interstitial molecular Cl_2_, (**c**) complex consisting of four Si–Cl groups, (**d**) interstitial atomic chlorine, and (**e**) SiCl_2_ arrangement. Reprinted with permission from Ref. [42]. Copyright 2024 American Chemical Society.

Chlorine has been reported to be contained in amorphous silica particles in various forms, as shown in Figure 11. Among them, the ≡Si–Cl bond (Figure 11a) is considered to be the most common structure [170]. Here, the ≡Si–Cl bond has been reported to form the weak hydrogen bond, which was observed in the FT-IR spectra at 2810 cm^−1^ [171,172].

**Figure 12:**
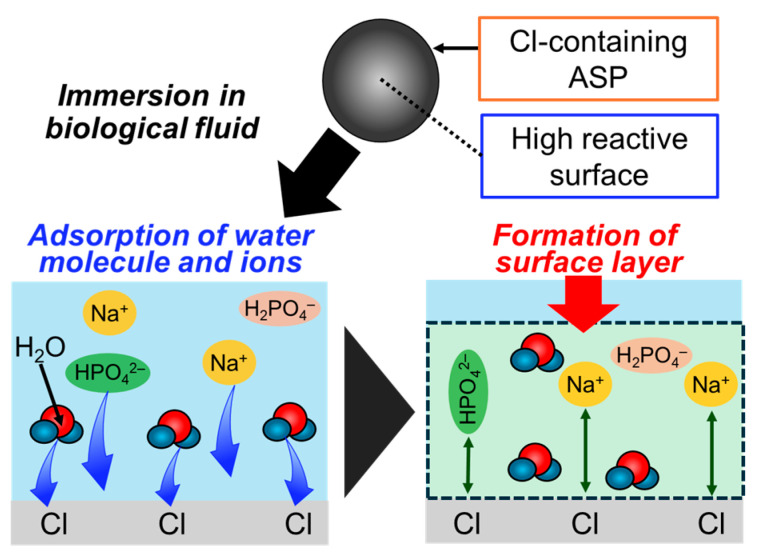
Illustration of the adsorption processes of the hydration layer on bioceramics and subsequent protein adlayer on the surface.

The molecular Cl_2_ in the interstitial lattice (Figure 11b) [173], the complexes consisting of the four ≡Si–Cl bonds known as the defect centers (Figure 11c) [174], the atomic Cl in the interstitial lattice (Figure 11d) [174], and the SiCl_2_ bond defects formed by the bonding to the two-coordinated silicon atoms (Figure 11e) [175] have been reported. Particularly, the ≡Si–Cl bonds on the surfaces can enhance the adsorption of water molecules and ions in biological fluids. Specifically, the ≡Si–Cl bonds are highly electronegative and can form weak hydrogen bonds. Then, the adsorption of Na^+^ and phosphate ions from biological fluids is thought to enhance the formation of the surface layer (Figure 12) [171,172]. As a result, the adsorption of water molecules and ions would be enhanced as compared to the case in conventional amorphous silica particles, leading to the formation of a surface layer similar to the non-apatitic layer and a steric immobilization state of proteins. In fact, the surface layer composed of water molecules and ions formed on the chlorine-containing silica particles in our group, leading to an increase in the amount of immobilized protein. Additionally, the protein was successfully immobilized while preserving its highly ordered structure [42].

## 5. Conclusions

In this review article, the research on the surface design of bioceramic particles is described and highlighted. The studies on the relationship between the surface properties of bioceramic particles and the protein immobilization states were deepened. We hope that the surfaces of bioceramic particles can effectively act on proteins and cells, which can be applied to DDS and achieve the phenomenon of ultra-early tissue regeneration. Furthermore, this technology can also be applied to the surface design of bioceramics for bone regenerative therapy. Particularly, osteoblasts recognize and adhere to the pre-immobilized cell-adhesive proteins in maintaining their highly ordered structures, indicating the importance of the studies on the interfacial properties between the bioceramic particle surfaces and proteins. In the future, research on surface design with positive effects on biological tissues is expected to contribute to the construction of social infrastructure that promotes health and vitality.

## Data Availability

Not applicable.
